# Increased Serum Levels of Tumor Necrosis Factor-like Ligand 1A in Atopic Dermatitis

**DOI:** 10.3390/ijms24031813

**Published:** 2023-01-17

**Authors:** Teruyoshi Hisamoto, Hiraku Suga, Asako Yoshizaki-Ogawa, Shinichi Sato, Ayumi Yoshizaki

**Affiliations:** Department of Dermatology, Graduate School of Medicine, University of Tokyo, Tokyo 113-8654, Japan

**Keywords:** AD, TL1A, innate lymphoid cells

## Abstract

Atopic dermatitis (AD) is a common chronic skin disease with pruritus, affecting 5–20% of the population in developed countries. Though its cause varies from genetic polymorphisms to the environmental factors, the T-helper (Th) 2 inflammation is one of the main characteristic pathoses. TNF superfamily ligand A (TL1A) is a recently discovered cytokine, which is released by various immune cells and reported to have an ability to stimulate Th1, Th2, and Th17 responses. Its association was investigated in chronic inflammatory disease, such as rheumatoid arthritis, inflammatory bowel disease, and psoriasis. However, its role on AD is unclear. To elucidate the association of TL1A in AD, we measured the serum TL1A levels in AD patients and healthy controls and performed the immunohistochemistry of TL1A. The result showed that the serum TL1A levels were higher in AD patients than healthy controls, and they positively correlated with the serum immunoglobulin E levels, serum Lactate dehydrogenase, and the number of eosinophils in peripheral blood. The immunohistochemistry of TL1A also showed TL1A expression in epithelium of AD samples. Because previous studies indicate TL1A has a certain role as an inflammation enhancer in Th2 and/or Th17 polarized disease, TL1A in AD may also has a role as an inflammation generator.

## 1. Introduction

Atopic dermatitis (AD) is a common skin disease characterized by repeated eczema with pruritus [1,2]. Its prevalence varies by country and by age, affecting 5–20% of the population in developed countries [1,2]. Its pathogenesis has been studied, and some genetic predisposition and environmental cause is known. As a genetic one, the disruption of the epidermal barrier molecules such as filaggrin, loricrin, and involucrin is reported [3,4,5]. In addition, allergy to various substances and subsequent type 2 inflammation also contribute to the development of AD [1,6]. The type 2 cytokines, interleukin (IL)−4 and IL−13, play a central role in the type 2 inflammation. These cytokines are released mainly by activated T-helper 2 lymphocytes (Th2) and innate lymphoid cells (ILC), which induce the proliferation of T lymphocytes and regulation of Th2 environment, as well as induce the production of the immunoglobulin E (IgE) by B lymphocytes [7]. Moreover, these cytokines are known to downregulate the expression of filaggrin, loricrin, involucrin, and lipid components of the skin barrier in the keratinocytes, resulting in further inflammation [4]. Now, based on this knowledge, the anti-IL−4 receptor alpha antibody (ab), dupilumab, and orally administrated Janus kinase inhibitors, which block the downstream intracellular signaling of IL−4 and IL−13, are shown to have the significant clinical effect on severe AD patients, bringing breakthrough progress to the treatment of AD [8,9,10]. Recently, the involvement of T helper 17 cells (Th17), which produce IL17, has also been reported in AD patients [11,12,13]. Th17 is activated in the skin rash area and peripheral blood, and it releases inflammatory cytokines, such as IL−17A, IL−17F, IL−22, and IL−26, to contribute to the pathogenesis of the disease [14]. Th17 activation is not seen in all AD patients, but in a part of them, with higher prevalence in Asia compared with in Europe [15,16].

On another front, tumor necrosis factor-like protein 1A (TL1A) is a member of the tumor necrosis factor superfamily of ligands, first described in 2002 [17]. TL1A is a type 2 transmembrane protein and is subsequently released as a soluble form, exerting pleiotropic effects on cell proliferation, activation, and differentiation of immune cells [18]. Though TL1A was first reported as an endothelial factor, now it is revealed to be expressed by lymphocytes, macrophages, and dendric cells [19]. As a receptor of TL1A, death receptor 3 (DR3) and decoy receptor 3 (DcR3) have been only identified [20]. DR3 is mainly expressed by CD4+ T cells and natural killer cells, which activates inflammatory signaling pathways, in both the innate immune system and the adaptive immunity [19,21]. The other receptor, DcR3, is a secreted protein that lacks the cytoplasmic domain and therefore works as a neutralizing receptor for TL1A. TL1A is shown to stimulate both Th1 and Th17 responses, and this new cytokine axis has been investigated in chronic inflammatory disorders such as rheumatoid arthritis or inflammatory bowel disease (IBD) [20,22,23]. 

With regard to skin disease, TL1A is involved in the pathogenesis of psoriasis, which is known as a representative Th17 dominant disease [24]. The expression of TL1A and DR3 is observed in the skin rash area, and upregulation of TL1A is also reported in peripheral blood mononuclear cells (PBMCs) in psoriasis patients though the mechanism of participation in psoriasis is not clarified yet [25]. Based on these above information, TL1A expression can be also upregulated in AD, since AD and bronchial asthma are both Th2 disease, and Th17 is also involved in AD, just as psoriasis. However, there are few reports and information about the association of TL1A in AD. In this article, we measured the serum TL1A levels in AD and healthy control, and analyzed the correlation between serum TL1A levels and clinical markers for AD. In addition, we also performed the immunohistochemistry of TL1A using the skin samples of AD and healthy control.

## 2. Results

### 2.1. Serum TL1A Level Was Elevated in AD Samples

**Figure 1:**
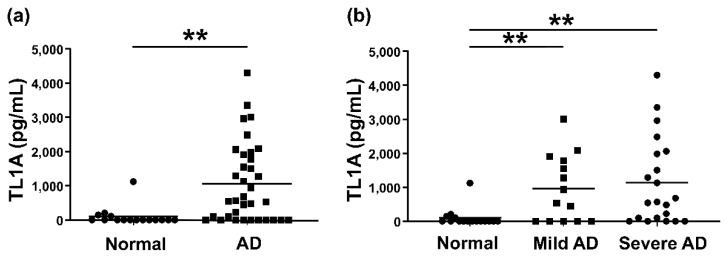
Serum TL1A protein levels in patients with AD and normal controls. The measured values from individual patients were plotted by dots. Each bar means the average. ** *p* < 0.01. (**a**) Serum TL1A protein levels in AD (n = 36) or normal controls (*n* = 10). (**b**) Serum TL1A levels in mild (*n* = 14) or severe (*n* = 22) AD patients and normal controls (*n* = 16).

To evaluate the association of TL1A in AD, we first measured serum TL1A concentration using enzyme-linked immuno-sorbent assay. Serum TL1A levels in AD samples were higher than those in healthy controls, although some samples did not have detective concentration (Figure 1a). Divided to severity, samples with severe AD, defined as Eczema Area and Severity Index (EASI) > 20, tended to have higher TL1A levels, but statistical significance was not seen between mild AD (EASI < 20) and severe AD (Figure 1b).

### 2.2. Correlation between TL1A and Clinical Marker for AD

**Figure 2:**
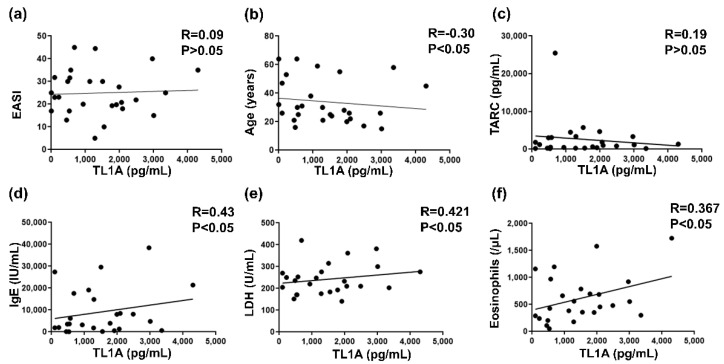
Correlation between TL1A protein levels and clinical markers in TL1A positive patients of AD. (**a**) Correlation between serum TL1A protein levels and Eczema Area and Severity Index (EASI). (**b**) Correlation between serum TL1A levels and the age of each patient. (**c**) Correlation between serum TL1A levels and serum thymus and activation-regulated chemokine (TARC) levels. (**d**) Correlation between serum TL1A levels and serum immunoglobulin E (IgE) levels. (**e**) Correlation between serum TL1A levels and serum lactate dehydrogenase (LDH) levels. (**f**) Correlation between serum TL1A levels and the number of eosinophils in peripheral blood.

We next analyzed the correlation between serum TL1A levels and clinical markers for AD. The age, EASI, serum thymus, and activation-regulated chemokine (TARC) levels, serum IgE levels, serum lactate dehydrogenase (LDH), and the number of eosinophils in peripheral blood were analyzed (Figure 2). As a result, the serum TL1A levels had positive correlations between LDH, IgE, and the number of eosinophils with statistical significance. In contrast, serum TL1A levels negatively correlated to the age of each patient though the correlation was weak. EASI and TARC had no correlation to TL1A. 

### 2.3. Involvement of Bronchial Asthma in AD Samples

**Figure 3:**
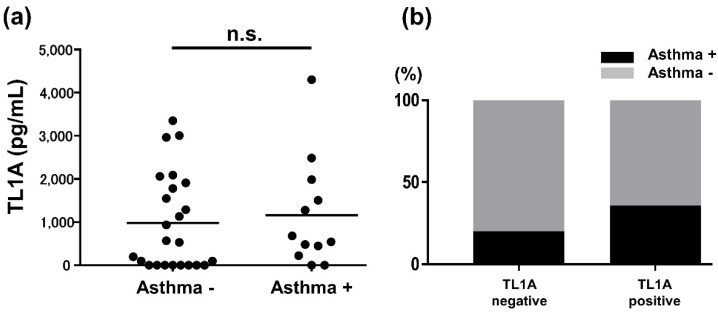
The association of bronchial asthma in the analysis samples. (**a**) Clinically, the patients were divided into two groups: those with asthma or with pre-existing asthma (asthma + ) and those without pre-existing or comorbid asthma (asthma-). (**b**) Whether each sample corresponds to Asthma+ or Asthma- group was analyzed in TL1A positive and negative group respectively.

We investigated the dependence of bronchial asthma (BA) to confirm that the serum TL1A levels in AD patients were elevated due to BA. In total of 36 AD samples, 12 patients had a history or were under treatment of BA (Figure 3). Out of 28 TL1A positive samples, 10 patients had BA history. Out of eight TL1A negative samples, two patients had BA history. As a result, there was no significance in serum TL1A concentration between the BA positive group and the BA negative one.

### 2.4. Immunohistochemistry

**Figure 4:**
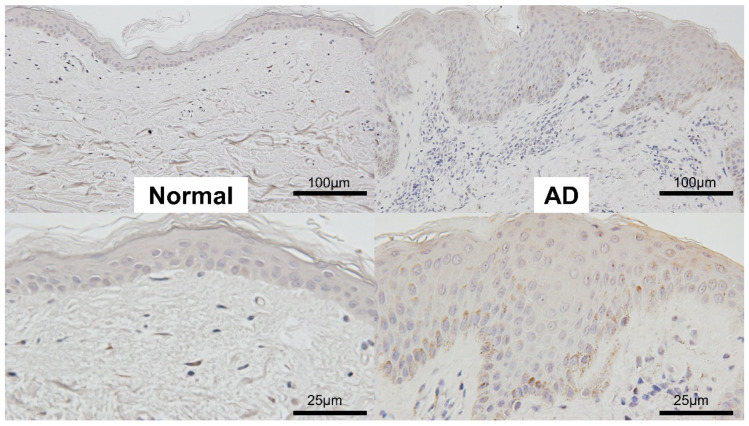
Immunohistochemistry of TL1A in atopic dermatitis (AD) or normal skin samples.

The immunohistochemical staining of TL1A revealed that epidermis in AD skin expressed TL1A to some extent. Representative photos were shown (Figure 4). In AD samples, eight were positive, five were weekly positive, and three were negative of the total of 16. In normal skin, two of ten were weekly positive and the others were negative. This result showed that TL1A was released mainly from keratinocytes in AD.

Immunohistochemistry was performed with anti-human TL1A antibody (ab234307, Abcam, UK). Representative photos were shown. Left two panels show normal skin, and right two panels show AD. Low magnification in upper panels, and high magnification in lower panels.

## 3. Discussion

First, we showed that serum TL1A levels were elevated in AD patients compared to healthy controls (Figure 1). They positively correlated with serum IgE levels, serum LDH levels, and eosinophil counts in the peripheral blood, although there was no statistical association with EASI or serum TARC levels (Figure 2). To see the association of BA, we tallied up the BA history of each patient, resulting in no significant dependence between TL1A levels and BA history (Figure 3). Furthermore, in immunohistochemistry, TL1A staining was virtually absent in normal specimens, but was generally positive in the basal cells of the epidermis, in many AD specimens (Figure 4).

In recent years, much attention has been paid to the role of ILC in AD. In skin, group 2 ILC (ILC2] recognize IL−25, IL−33, and thymic stromal lymphopoietin, cytokines released from damaged epithelia, inducing inflammation by releasing IL−5 and IL−13 [26,27,28]. Other various cytokines, including IL−4, have been reported to be involved in their activation. Unlike normal Th2 lymphocytes, ILC2 can produce amount of Th2 cytokines without antigen-dependent manner [29,30]. To date, it has been shown that, in BA, IBD, and rheumatoid arthritis, TL1A also stimulates ILC2 to form their disease condition, suggesting that TL1A also has the ability to activate the skin resident ILC2 [19,20,31]. Our series of results in this article is consistent with these insights. However, TL1A may not act directly on the skin lesion, but may be indirectly involved in the inflammation of AD. This is because serum TL1A levels did not well correlated with EASI or serum TARC levels, which are both commonly used to assess the severity of AD, but did correlate with IgE and eosinophil counts, both of which are indicators of allergy. Importantly, it is well known that BA, frequently accompanied by AD, also have Th2 polarized inflammation and characterized by high serum IgE levels [32,33]. Therefore, we verified the association of BA in our samples, resulting in no apparent relationship (Figure 3). The reason for the lack of the correlation between TL1A levels and EASI or TARC remains unknown. 

On the other hand, the effect of TL1A on Th17 have also been studied. As mentioned above, the TL1A expression is seen in the psoriatic skin plaques, and PBMCs with psoriasis also secrete TL1A [24,25]. TL1A expression is observed in keratinocytes and basal cells in the psoriatic skin lesions with hyperplasia, as well as in the perivascular inflammatory cells in the epidermis [24]. In vitro, TL1A, together with IL−23, promotes the release of IL−17, indicating that TL1A contributes to the maintenance of the Th17 environment [25]. Its receptor, DR3, is also reported to be expressed by PBMCs in psoriasis vulgaris, especially by CD8+ and CD14+ PBMCs [34]. Although the involvement of ILC in psoriasis has been reported, the relationship among Th17, TL1A, and ILC has not yet been clarified [35,36]. Additionally, not only Th2, but also Th17, are involved in AD, and some patients are Th17 dominant, and, interestingly, pediatric AD tends to be Th17-polarized [13]. The decoy receptor for TL1A and DcR3 is also upregulated in pediatric AD patients compared with healthy control or adolescent AD [37]. This information is consistent with our result that serum TL1A levels was negatively correlated to age (Figure 2). Therefore, relatively young AD patients may tend to have Th17 polarized phenotype and have high serum TL1A levels. These differences of phenotypes can explain the discrepancy with the previous report that serum TL1A levels in AD, as a disease control of psoriasis, tended to be increased compared with controls, without significant difference [25]. Furthermore, we have to note that Th17 polarization is also sometimes accompanied by high serum IgE levels [38,39]. Generally, IgE is acknowledged as a typical mediator of allergic reaction and is elevated in AD [40]. Our result of the positive correlation between serum TL1A levels and serum IgE levels can be explained simply by AD disease severity, but also by the relationship among IgE, TL1A activation and Th17 polarization. These complicated relationship among TL1A-associated natural immunity and Th2- and/or Th17- mediated acquired immunity is not clarified yet. 

Though no therapy targeting TL1A is clinically used in general, experiments in animal models have shown that blocking TL1A by the inhibition with antibody or using transgenic mice improved asthma and IBD [41]. Actually, a phase 2a study of a fully human immunoglobulin G1 monoclonal antibody has been performed, revealing a good tolerance and effect on IBD [42]. As the current biological therapies used in AD are targeted to IL−4/IL−13 pathway, the inhibition of TL1A may have the independent and new therapeutic value for the AD treatment.

In our study, the precise mechanism of TL1A elevation and which cells mainly release TL1A in AD remain unclear. The association between TL1A and proinflammatory cytokines, such as IL−25, IL−33, or TLSP, is also unclear. Oppositely, just as IL−4, TL1A may have the ability to downregulate the epidermal barrier molecules, such as filaggrin, loricrin, and involucrin. It is necessary to further elucidate the pathogenesis and the positioning of TL1A, which can explore the possibility of clinical application.

## 4. Materials and Methods

### 4.1. Clinical Samples

Serum samples were obtained from 36 patients with AD and 16 healthy control subjects in our department. The profiles of the samples were shown in Table 1. Clinical and laboratory data of these AD patients are shown in Table 2. Samples for immunohistochemistry were collected from AD patients (*n* = 16) and normal skin adjacent to benign skin tumors (*n* = 8). All samples were collected after informed consent during daily clinical practice. The medical ethical committee of the University of Tokyo approved all described studies (0695–20), and the study was conducted according to the Declaration of Helsinki Principles. 

### 4.2. Enzyme-Linked Immunosorbent Assay

Serum TL1A levels were quantified using Human TL1A ELISA kit (DY1319–05 and DY008, R&D Systems, Minneapolis, MN, USA). These assays employ the quantitative sandwich enzyme immunoassay technique. Optical densities were measured at 450 nm with the correction wavelength set at 570 nm, using a Bio-Rad Model 550 microplate reader (Bio-Rad Laboratories, Hercules, CA, USA). The concentrations were calculated from the standard curve generated by a curve-fitting program. The detection limit of TL1A was set at 20 pg/mL.

### 4.3. Immunohistochemistry

Immunohistochemical staining for TL1A was performed in normal skin adjacent to benign skin tumors as healthy controls (*n* = 10) and lesional skin of AD (*n* = 16). Briefly, 5-μm thick tissue sections from formaldehyde-fixed and paraffin-embedded samples were dewaxed and rehydrated. After the antigen retrieval by Tris ethylenediaminetetraacetic acid Buffer, pH 9.0 (Agilent, Santa Clara, CA, USA), these sections were stained with 2 μg/mL of rabbit anti-human TL1A monoclonal Ab according to the manufacturer’s protocol (ab234307, Abcam, Cambridge, UK), followed by ABC staining (Vector Lab, Newark, CA, USA). Diaminobenzidine was used for visualizing the staining, and counterstaining with Mayer haematoxylin was performed, according to the manufacturer’s instructions. The positivity of staining was ranked from negative, weekly positive, to positive.

### 4.4. Statistical Analysis

Statistical analysis was performed using the Mann-Whitney U-test for two groups. Correlation coefficients were determined using Spearman’s rank correlation test. Fisher’s exact test was used in the analysis of contingency tables. In each test, a *p*-value < 0.05 was considered statistically significant.

## Figures and Tables

**Table 1:** Characteristics of human samples.

Characteristics	Normal	AD
Number	16	36
Age(years)	39.5 ± 15.5	36.3 ± 16.2
Sex (male/female)	10/6	26/10

**Table 2:** Clinical and laboratory data of AD patients.

EASI score	24.8 ± 10.1
TARC (IU/mL)	2413 ± 4361
IgE (IU/mL)	7877 ± 9770
LDH (U/L)	240.5 ± 67.9
Eosinophils (/uL)	540 ± 396

EASI: Eczema Area and Severity Index, TARC: Thymus and activation-regulated chemokine, IgE: Immunoglobulin E, LDH: Lactate Dehydrogenase.

## Data Availability

Not applicable.
